# Evaluating Spatial-Temporal Dynamics of Net Primary Productivity of Different Forest Types in Northeastern China Based on Improved FORCCHN

**DOI:** 10.1371/journal.pone.0048131

**Published:** 2012-11-07

**Authors:** Junfang Zhao, Xiaodong Yan, Jianping Guo, Gensuo Jia

**Affiliations:** 1 Chinese Academy of Meteorological Sciences, Beijing, People’s Republic of China; 2 State Key Laboratory of Earth Surface Processes and Resource Ecology (ESPRE), College Of Global Change and Earth System Science, Beijing Normal University, Beijing, People’s Republic of China; 3 Institute of Atmospheric Physics, Chinese Academy of Sciences, Beijing, People’s Republic of China; DOE Pacific Northwest National Laboratory, United States of America

## Abstract

An improved individual-based forest ecosystem carbon budget model for China (FORCCHN) was applied to investigate the spatial-temporal dynamics of net primary productivity of different forest types in northeastern China. In this study, the forests of northeastern China were categorized into four ecological types according to their habitats and generic characteristics (evergreen broadleaf forest, deciduous broadleaf forest, evergreen needleleaf forest and deciduous needleleaf forest). The results showed that distribution and change of forest NPP in northeastern China were related to the different forest types. From 1981 to 2002, among the forest types in northeastern China, per unit area NPP and total NPP of deciduous broadleaf forest were the highest, with the values of 729.4 gC/(m^2^•yr) and 106.0 TgC/yr, respectively, followed by mixed broadleaf- needleleaf forest, deciduous needleleaf forest and evergreen needleleaf forest. From 1981 to 2002, per unit area NPP and total NPP of different forest types in northeastern China exhibited significant trends of interannual increase, and rapid increase was found between the 1980s and 1990s. The contribution of the different forest type’s NPP to total NPP in northeastern China was clearly different. The greatest was deciduous broadleaf forest, followed by mixed broadleaf- needleleaf forest and deciduous needleleaf forest. The smallest was evergreen needleleaf forest. Spatial difference in NPP between different forest types was remarkable. High NPP values of deciduous needleleaf forest, mixed broadleaf- needleleaf forest and deciduous broadleaf forest were found in the Daxing’anling region, the southeastern of Xiaoxing’anling and Jilin province, and the Changbai Mountain, respectively. However, no regional differences were found for evergreen needleleaf NPP. This study provided not only an estimation NPP of different forest types in northeastern China but also a useful methodology for estimating forest carbon storage at regional and global levels.

## Introduction

As a key variable in our understanding of ecosystem processes and carbon exchange between biota and atmosphere, both currently and under climate change scenarios [1], net primary productivity (NPP) is defined as the difference between accumulated photosynthesis and accumulated autotrophic respiration by green plants per unit of time and space [2]. Relative to other ecosystems, forest ecosystems play an important role both in global and regional carbon cycles regulation because of their larger carbon stocks, sequestration capacity, and productivity [3], [4], [5], [6]. A slight change in NPP of forests can significantly influence atmospheric CO_2_ concentration and, consequently, climate change. Therefore, it is important to quantify carbon storage and fluxes for different forest types and analyze mechanisms involved in carbon cycling to better monitor the processes that regulate the uptake, storage, and release of CO_2_ [7].

In China, forests are mainly composed of young to middle-aged secondary forests and plantations [8], and therefore, the ecosystem′s carbon cycle is far from being stabilized. Hence, quantification of forest carbon cycle is an important part of national inventories of net greenhouse gas emissions in a country [9], [10]. The forest ecosystems in northeastern China play an important role in the national carbon budget because they comprise more than 30% of the total forest area [11] and 40% of the total forest biomass of China [6], including the southern boundary boreal forests of Eurasia in the Da Hinggan Mountains of the Heilongjiang Province and Inner Mongolia, which are especially sensitive to projected climate change [12]. In the past decade, large-scale NPP characteristics of forest ecosystems have been studied based on different methodologies such as national forest inventory data [13]–[18] and process-based terrestrial biosphere models [1], [19], [20], [21]. The results showed that China’s terrestrial NPP has been significantly increased due to the increases in temperature, precipitation, and CO_2_ concentrations, and the largest increases in NPP were observed in broad-leaf and needle-leaf mixed forests to the northeast of China [14], [20]. The average forest NPP, which was investigated the spatio-temporal changes of the forest′s NPP in China over the recent two decades based on a geographically weighted regression (GWR) with a cumulative remote sensing index, was essentially unchanged from the 1980s to late 1990s [6]. These previous studies are important for understanding various aspects of forest dynamics, NPP, and carbon balance; however, because of difficulties in obtaining long-term observations of disturbances, very limited studies have been conducted to quantitatively investigate the spatial-temporal trends of NPP of different forest types in northeastern China at a regional scale so far.

Getting a clear picture of the NPP of ecosystems at the regional or global scales through direct measurements is very difficult. Therefore, ecological models have practically become one of the best approaches that integrate all available data sets for large-scale applications for investigating the terrestrial carbon cycle [12]. Process-based models are increasingly used to better understand how the key processes that govern the dynamics of the forest ecosystems interact. For example, an individual-based forest ecosystem carbon budget model for China (FORCCHN) [22] has been used in investigating the forest carbon cycle of China.

The objective of this study was to 1) investigate the spatial-temporal distribution of NPP of different forest types in northeastern China by taking advantage of the improved FORCCHN model, to 2) simulate the dynamic mechanism of carbon cycle of young forests, and 3) provide scientific evidence for reasonably estimating carbon sequestration and the dynamics of different forest types in northeastern China under climate change.

## Materials and Methods

### 1. Study Area

**Figure 1:**
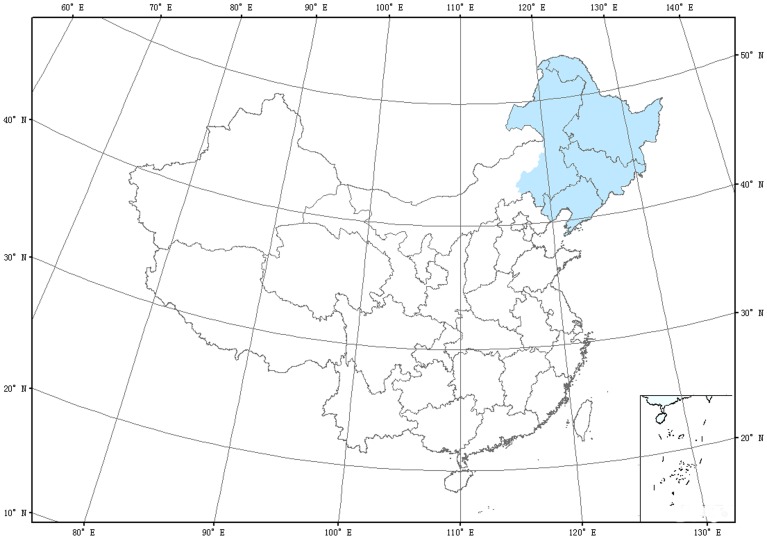
The location of the study region in China.

The forest areas of the northeastern China are located in the southern edge of the Eurasia boreal forests, including Heilongjiang, Jilin, and the Liaoning provinces, and the eastern part of Inner Mongolia Autonomous Region (Figure 1). The study area covers about 1 240 000 km^2^ (about 12.9% of the total country′s area) ranging from 38**–**54°N and from 110**–**136°E. The forests in northeastern China represent a transition zone between boreal and temperate vegetation, and therefore is likely to be sensitive to the changes in climate and play an important role in the carbon cycle in East Asia [12], [23]. There are four dominant forest types, including temperate evergreen needleleaf forests (*Picea*, *Pinus*), deciduous needleleaf forests (*Larix gmelinii*), deciduous broadleaf forests (*Robinia pseudoacacia*), and mixed forests (*Pinus koraiensis*) [12], [24]. Major soil types include dark-brown soil, brown needleleaf forest soil, calcic chernozems and meadow soil. The climate in this study area is characterized by warm summer, cold winter, abundant precipitation, and short growing season, largely controlled by the East Asian monsoon, changing from a warm temperate zone to a cool temperate zone from south to north, and from a humid zone to a semiarid zone from east to west. For the whole study area, the average annual temperature ranges from –4°C to 11.5°C and the annual precipitation ranges from 250 mm in the west to 1100 mm in the east [25].

### 2. FORCCHN Model and its Optimization

**Figure 2:**
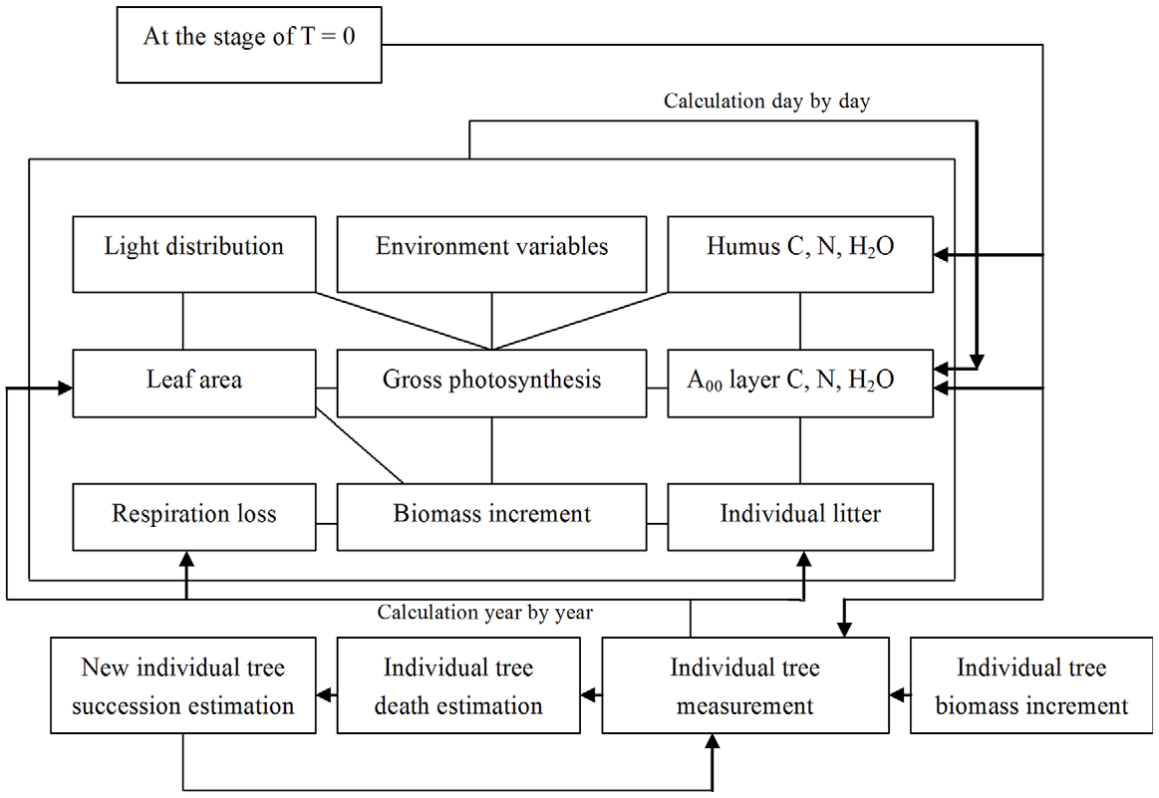
The primary processes and flow charts of FORCCHN model.

The carbon budget model FORCCHN was established based on individual tree species [22], and further improved by Zhao *et al.* [12]. The model is driven by daily meteorological data, soil characteristics data, and vegetation characteristics data. And it is aimed to simulate carbon budget of individual trees in given study areas. Through summing or coupling soil carbon cycle model, the FORCCHN model can be used to calculate carbon cycle of forest ecosystems in unit area. The major processes considered in the model and the flow charts are illustrated in Figure 2. The FORCCHN model is built with four major characteristics in mind. Firstly, the carbon, water, and nutrient cycles must be fully coupled in the soil-plant-atmosphere system. Secondly, the external constraints on the model’s behavior and driving forces for the model must be the same as those which operated based on individuals. That is, the constraints must, as far as possible, be fundamental biological and physico-chemical processes and the driving variables can not be decided by current climate and statistical relations of ecosystems in advance. Thirdly, the carbon budget of ecosystem is decided by the growth of individuals in a stand, therefore it can be estimated with reasonable accuracy. Fourthly, the model must be capable of predicting dynamic processes of carbon budget of forest ecosystem induced by climate change and equilibrium responses to climate change in the future.

#### Major control equations of the FORCCHN Model

The carbon budget equations of individual tree and forest stands are calculated as follows [22]:
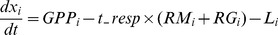
(1)

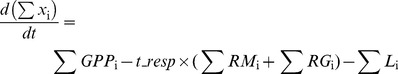
(2)where 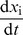 denotes the daily carbon budget increment of ith individual tree (i = 1, 2, …, n)(t = 0, 1, 2, …, n) (kgC/d); 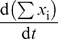, the daily carbon budget increment of a forest stand (kgC/d); *GPP*_i_, the daily gross primary productivity of ith individual tree (kgC/d); *RM*_i_, the daily maintenance respiration of ith individual tree (kgC/d); *RG*_i_, the daily growth respiration of ith individual tree (kgC/d); *L*_i_, the daily litter amount of ith individual tree (kgC/d), and *t_resp*, the effect of air temperature on plant respiration that ranges from 0 to 1.

#### Primary daily processes

The primary daily processes include photosynthesis, plant respiration, allocation and litter production, and soil respiration and transfer. It is assumed that the *i*th individual belongs to *j*th plant function type.

Photosynthesis:Gross primary productivities of each individual tree and stands in unit area are given by:
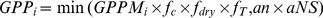
(3)

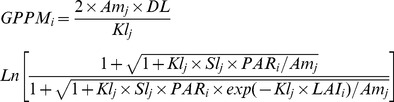
(4)


Where *GPP_i_* is the daily gross primary productivity of each individual (kgC/d), *GPPM_i_* the daily gross primary productivity of stand (kgC/d), *DL* the possible sunshine duration (h), *PAR_i_* the photosynthetic active radiation of canopy at noon (W/m^2^), *Am_j,_* the possible maximal leaf photosynthesis [kgC/(m^2^·h)], *Kl_j_* the extinction coefficient, *Sl_j_* the initial slope of light intensity and photosynthesis [(kgC/(m^2^·h))/(W/m^2^)], *LAI_i_* the leaf area index, *f_c,_* the effect of carbon dioxide on gross primary productivity, *f_dry_* the effect of water on gross primary productivity, *f_T_* the effect of temperature on gross primary productivity, an×*aNS* the effect of soil active nitrogen on gross primary productivity; *aNS* the soil active nitrogen amount (kgN/m^2^), and an = 150.

Respiration:The autotrophic respiration of plant includes maintenance respiration and growth respiration. The formulas for maintenance respiration and growth respiration are expressed as:
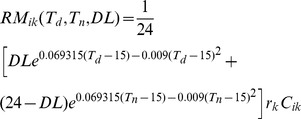
(5)

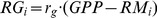
(6)


Where *RM_ik_* is daily maintenance respiration (kgC/d); *T_d_* the daily average air temperature (°C); *T_n_* the mean night-time air temperature (°C); *DL* the possible sunshine duration (h); r_k_ the relative respiration rate of foliages, branches, stems, main roots, and fine roots at 15°C (1/d); *C_ik_* the carbon pool amount (kgC), and when *k* denotes leaves or fine root, *C_i_* is leaf content or fine root content; When *k* denotes stem or root, *C_ik_* is sapwood content (kgC); *RG_i_* the daily growth respiration (kgC/d); *r_g_* the growth respiration coefficient, and r_g_ = 0.25; *GPP* the daily gross photosynthesis (kgC/d).

Litter production:The litter fluxes of leaves and fine root are computed as follows:
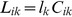
(7)


Where *L_ik_* is litter fluxes of foliage or fine root (kgC/d); *C_ik_* the corresponding carbon pool (kgC/d); *l_k_* the relative littering rate (1/d).

Allocation:According to the allocation mechanism of photosynthesis production, the model assumes that net photosynthesis production is only partitioned to leaves, fine roots and litters, while other photosynthesis productions are stored in a so-called buffer carbon pool in daily processes. Therefore the increments △C_ik_ of leaves and fine roots and the possible maximal leaf carbon content or fine root carbon content are:
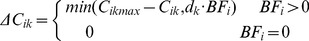
(8)

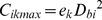
(9)


Where *BF_i_* is daily buffer carbon pool of the number *i*th tree (kgC), *d_k_* the partition proportion coefficient, *d_fine root_*+*d_leaf_* = 1, *e_k_* the proportion coefficient (kgC/m^2^), *D_bi_* the diameter at breast height (m).

Soil organic matter respiration and transfer progress: the model assumes that soil processes are at daily time scale, and therefore adopts a modified soil carbon budget model CENTURY to characterize forest soils. The CENTURY model was originally developed for simulating and forecasting carbon cycle and productivity of grassland, but now it is widely used for forest ecosystems. Through simulating the biology geochemistry cycle of carbon, nitrogen and phosphorus and some driving factors such as temperature and precipitation, it is capable of predicting productivity of ecosystems.

The major processes and formulas in the modified CENTURY model [26] are described as follows:

Leaf litter and fine root litter are sub-divided between soil structural and metabolic litter pool and the proportions are:
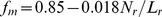
(10)

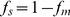
(11)


Where *f_m_* is the proportion of fresh litter classified as metabolic, *f_s_* the proportion classified as structural litter, *N_r_* and *L_r_* are the respective concentrations of nitrogen and lignin in fresh litter.

The decomposition rate, respiration release and carbon transported in other carbon pool are calculated as:
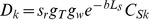
(12)

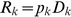
(13)

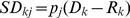
(14)

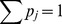
(15)

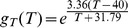
(16)

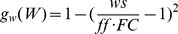
(17)


Where *D_k_* is daily decomposition amount of the number *k*th carbon pool [kgC/(m^2^·d)]; *s_r_* the referenced relative decomposition rate (1/d); *G_t_* and *g_w_* the coefficient described the effect of temperature and water on the decomposition rate; b is constant value of 5.0; *L_s_* the lignin content in metabolic litter, and otherwise 0; *C_sk_* the difference of soil carbon pool and soil lignin pool (kgC/m^2^); *R_k_* the respiration release amount [kgC/(m^2^·d)]; *p_k_* the proportion of respiration; *SD_kj_* total carbon transported [kgC/(m^2^·d)]; *p_j_* the proportion transported to *j*th carbon pool; *ws* the water content of soil or litter (cm); *f* is constant, 0.6; *FC* the field holding capacity (cm); *T* the soil temperature (°C).

#### Primary annual processes

The primary annual processes consist of allocation between stands, increase of tree height, DBH, and production of large amount of litter fall. Every year carbon cycled through non-individual death, such as litter production (including flower and fruit). There are two thresholds: if buffer carbon pool of each individual at the end of every year is bigger than the first threshold, the litter production of flower is maximal. If buffer carbon pool of each individual at the end of every year is greater than the second threshold, the litter productions of flower and fruit are maximal. The formulas are given as:



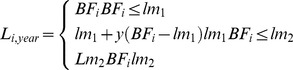
(18)

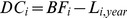
(19)


Where *L_i,year_* is litter production in a given year (kgC); *BFi* the buffer carbon of individual at the end of the year (kgC); *l_m_*_1_and *l_m2_* the first and the second thresholds, respectively (kgC); *DCi* the carbon store increment (kgC), changed by 95% of its carbon amount into other organs including the increase of tree height, basal diameter, height and diameter of branches. *DCi* also decides sapwood amount and possible maximal leaf area index or wood respiration in the next year.

Wood increment, basal diameter increment and height increment can be expressed as:
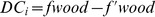
(20)

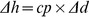
(21)

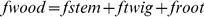
(22)

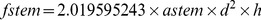
(23)

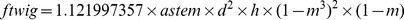
(24)

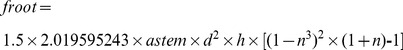
(25)


Where *DC_i_* is wood increment (kgC); *fwood* the wood biomass in the last year (kgC); *f’wood* the wood biomass in current year (kgC); *fstem* the stem biomass (kgC); *ftwig* the twig biomass (kgC); *froot* the root biomass (kgC); *d* the basal diameter (m); *△d* the increment of basal diameter (m); *h* the tree height (m); *△h* the increment of tree height (m); *b* the twig height (m); *hr* the root depth (m); *astem* the bulk density of wood (kgC/m^3^); *cp* is a constant decided by illumination grads of tree canopy; *m = b/h; n = hr/h*.

### 3. FORCCHN Model Input and Run

The model input is simply designed to include three parts, namely daily meteorological data, soil characteristics data, and vegetation characteristics data. The meteorological data are provided by the China Meteorological Administration. The soil characteristic data are from 1∶1 4000000 soil texture maps generated by the Institute of Soil of Science of Chinese Academy of Sciences. The vegetation characteristic data include maximal leaf area index, minimum leaf area index and forest cover. Since there are no direct ground measurements of forest leaf area indexes, the initial forest leaf area indexes are calculated through the NOAA satellite AVHRR NDVI data sets. NDVI data sets are derived from National Oceanic and Atmospheric Administration/Advanced Very High Resolution Radiometer (NOAA/AVHRR) satellite images.

The model parameters consist of soil parameters and tree type parameters. The forest are categorized into four ecological types according to their habitats and generic characteristics (evergreen broadleaf forest, deciduous broadleaf forest, evergreen needleleaf forest, deciduous needleleaf forest), such as shade tolerance (climax) species and sun tolerance (pioneer) species in northeast China. And they are all natural forests or plantations with more than 30% canopy densities and 2 m height. The soil parameters include soil organic matter parameters, litter pool decomposition parameters and soil physical parameters, and the soil physical parameters are principally dependent on geography position. The basic parameters of soil carbon cycle and the physiological and ecological parameters are shown respectively in Table 1 and Table 2.

**Table 1:** Parameters of soil decomposition in the FORCCHN model.

Symbol	Unit	Carbon pool	Value
S_1_	d^−1^	Above-ground metabolic litter pool	0.021
S_2_	d^−1^	Above-ground structural litter pool	0.1
S_3_	d^−1^	Below-ground metabolic litter pool	0.027
S_4_	d^−1^	Below-ground structural litter pool	0.13
S_5_	d^−1^	Fine woody litter pool	0.01
S_6_	d^−1^	Coarse woody litter pool	0.002
S_7_	d^−1^	Below-ground coarse litter pool	0.002
S_8_	d^−1^	Active soil organic matter pool	0.042
S_9_	d^−1^	Slow soil organic matter pool	0.001
S_10_	d^−1^	Resistant soil organic matter pool	3.5×10^−5^

The parameters are calculated according to the literature [26].

**Table 2:** Physiological and ecological parameters in the FORCCHN model.

Physiological and ecological parameters	Evergreen broadleaf species	Evergreen broadleaf species	Deciduous broadleaf species	Deciduous broadleaf species	Evergreen conifer species	Evergreen conifer species	Deciduous conifer species
	Shade tolerance	Sun species	Shade tolerance	Sun species	Shade tolerance	Sun species	
L_o_	5.5	11.0	5.5	11.0	5.5	11.0	11.0
Am	5.5×10^−4^	5.5×10^−4^	5.0×10^−4^	5.0×10^−4^	5.0×10^−4^	5.0×10^−4^	5.0×10^−4^
Sl	1.3×10^−5^	1.3×10^−5^	1.3×10^−5^	1.3×10^−5^	1.3×10^−5^	1.3×10^−5^	1.3×10^−5^
Kl	4.5×10^−1^	4.5×10^−1^	4.0×10^−1^	4.0×10^−1^	4.0×10^−1^	4.0×10^−1^	3.5×10^−1^
r_L_	2.0×10^−3^	2.0×10^−3^	6.0×10^−3^	3.0×10^−3^	3.5×10^−3^	3.5×10^−3^	1.2×10^−2^
r_W_	1.0×10^−3^	1.0×10^−3^	2.0×10^−3^	2.0×10^−3^	2.0×10^−3^	2.0×10^−3^	2.0×10^−3^
r_R_	1.5×10^−3^	1.5×10^−3^	2.5×10^−3^	2.5×10^−3^	2.5×10^−3^	2.5×10^−3^	2.5×10^−3^
lm_2_	0.40	0.40	0.40	0.40	0.50	0.50	0.50
CN_L_	45.0	45.0	40.0	40.0	60.0	60.0	50.0
CN_W_	200.0	200.0	200.0	200.0	200.0	200.0	200.0
CN_R_	45.0	45.0	40.0	40.0	60.0	60.0	50.0
Hmax	50.0	40.0	40.0	40.0	60.0	60.0	50.0
Dmax	2.0	1.5	2.0	1.5	2.0	2.0	2.0
Amax	400.0	200.0	400.0	200.0	1000.0	300.0	500.0
e_L_	600.0	600.0	200.0	700.0	700.0	700.0	300.0
e_R_	20.0	20.0	30.0	30.0	15.0	15.0	28.0
cLAI_L_	15.0	15.0	45.0	20.0	18.0	18.0	40.0
Astem	350.0	350.0	350.0	350.0	350.0	350.0	350.0
Tmin	3.0	1.0	−1.0	−5.5	−5.5	−2.5	−5.5
Topt	27.0	25.0	23.0	20.0	18.0	23.0	16.0
Tmax	50.0	50.0	45.0	45.0	40.0	40.0	35.0
DRY	0.9	0.8	0.8	0.6	0.9	0.7	0.5
l_L_	2.0×10^−3^	2.0×10^−3^	1.1×10^−4^	1.1×10^−4^	2.0×10^−3^	2.0×10^−3^	1.1×10^−4^
Lr/Nr	40.0	40.0	30.0	50.0	80.0	80.0	50.0
l_R_	5.0×10^−5^	5.0×10^−5^	4.0×10^−5^	4.0×10^−5^	8.0×10^−5^	8.0×10^−5^	8.0×10^−5^

The parameters are calculated according to the literature [27]. *Lo* the photosynthesis compensate point; *Am* the Maximal photosynthesis; *Sl* the initial slope of light intension and photosynthesis[kgC/(m^2^·h)/(W/m^2^)]; *Kl* the extinction coeffinient; *rL* the relative breath rate of foliage (1/d); *rW* the relative breath rate of wood (1/d); *rR* the relative breath rate of root(1/d); *lm*2 the threshold value of fruit; *CNL* the C:N ratio of foliage; *CNR* the C:N ratio of wood; *CNR* the C:N ratio of root; *Hmax* the maximal tree height (m); *Dmax* the maximal tree diameter (m); *Amax* the maximal tree age (a); *eL* the coefficient of leaf content (kgC/m^2^); *eR* the coefficient of root weigh (kgC/m^2^); *cLAIL* the coefficient of leaf area (m^2^/kgC); *astem* the bulk density of wood (kgC/m^3^); *Tmin* the lowest temperature of photosynthesis (°C); *Topt* the optimum temperature of photosynthesis (°C); *Tmax* the highest temperature of photosynthesis (°C); *dry* the capability of enduring drought; *lL* the relative litter rate of leaves(1/d); *Lr*/*Nr* the ration of lignin and nitrogen content; *lR* the relative litter rate of root(1/d).

The major initial conditions and boundary conditions of this study are based on the 10 km×10 km grid database. The simulations and a variety of environmental factors are assumed to be uniform in every grid. The initial conditions include, from 1980 to 2002, daily maximal, minimum and average air temperature (°C) and precipitation (cm), relative humidity (%), wind speed (m/s) and total radiation (W/m^2^). The boundary conditions include soil field capacity (cm), bulk density (kgC/m^3^), carbon pool (kgC/m^2^), nitrogen pool (kgN/m^2^), soil water content (cm), sand content (%), silt content (%), clay content (%), maximal leaf area index (m^2^/m^2^) and minimum leaf area index (m^2^/m^2^) in 1980 and forest cover (%).

This simulation begins on January 1st, 1980, and simulates NPP of forest ecosystems step by step in every grid. The areas of different forest type are a result of forest type assignment. Land use change is not considered, and vegetation types are fixed across the entire run. The relationships between soil and forest vegetation type were not considered. The detailed descriptions of the FORCCHN model′s features, structure, mathematical representation, basic parameters, building strategy and validation were previously provided by Yan and Zhao [22].

### 4. FORCCHN Model Optimization

Forest’s hydrological effect is an important function of forest ecosystem. With dense forest canopy foliage, there are twice distribution processes of the atmospheric precipitation mainly through the canopy interception. When the precipitation reaches the canopy of forest, the first precipitation redistribution occurs. Part of the precipitation is intercepted by the canopy, and part of the precipitation reaches the ground surface across the canopy gaps. The second redistribution of precipitation reached the woodland take places. Part is underground infiltration, and part is surface runoff. Through adding variables and modules of precipitation (rainfall and snowfall) interception by tree crown, understory plants and litter, Zhao *et al*. further improved FORCCHN and applied it to estimate net carbon budget of forest ecosystems and its response to climate change in northeastern China [12]. The detailed descriptions of the improved FORCCHN model′s features, mathematical representation, building strategy and validation were previously provided by Zhao *et al*. [12]. This current study presents our first attempt to apply the improved FORCCHN model for studying the spatial-temporal dynamics of NPP of different forest types (evergreen broadleaf forest?deciduous broadleaf forest?evergreen needleleaf forest?deciduous needleleaf forest) in northeastern China from 1981 to 2002.

## Results

### 1. Temporal Patterns of NPP of Different Forest Types

**Figure 3:**
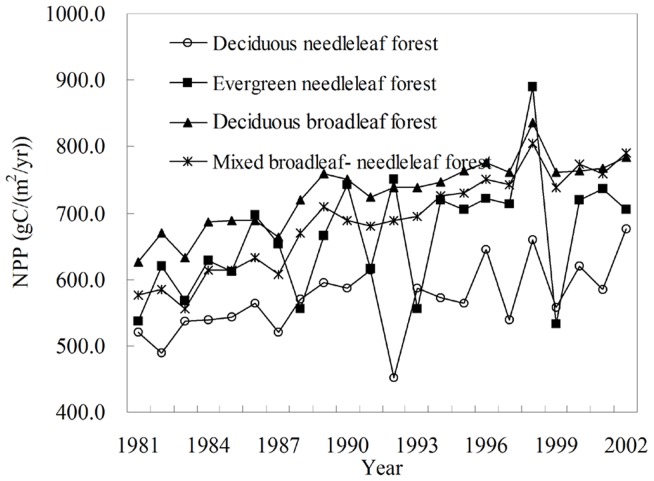
The interannual variation of per unit area NPP of different forest type in northeastern China from 1981 to 2002.

The temporal pattern of forest NPP was evident in the interannual variation. In northeastern China, per unit area NPP of different forest type from 1981 to 2002 exhibited significant trends of interannual increase, showing larger NPP fluctuation difference (Figure 3). A rapid increase was found between the 1980s and 1990s. And the biggest fluctuation appeared in evergreen needleleaf forest. Deciduous needleleaf forest and evergreen needleleaf forest behaved a lesser fluctuation. However, the NPP fluctuation of deciduous broadleaf forest was the smallest. It should be noted that the study period in this study was relatively short, and only across two decades. The interdecadal change in the long run was unstable. So, interdecadal change was analyzed based on a two sub-period of study period. The areas of different forest types in northeastern China were calculated and compared. Our result showed that the area of deciduous broadleaf forest was the biggest, with the value of 14.5 million ha, followed by mixed broadleaf- needleleaf forest and deciduous needleleaf forest, with the area value of 13.5 million ha and 10.3 million ha, respectively. Evergreen needleleaf forest occupied the smallest area, only 0.1 million ha.

Distribution and change of forest NPP in northeastern China were related with the different forest types. As for deciduous broadleaf forest, per unit area NPP was the biggest, with the average value of 729.4 gC/(m^2^•yr). The per unit area NPP of evergreen needleleaf forest and mixed broadleaf- needleleaf forest were higher than those of deciduous needleleaf forest and deciduous needleleaf forest, with the average value of 665.7 gC/(m^2^•yr) and 687.6 gC/(m^2^•yr), respectively. The deciduous needleleaf forest has the lowest per unit area average NPP of 569.7 gC/(m^2^•yr). The total NPP of different forest types in northeastern China was further analyzed. As for deciduous broadleaf forest, the total NPP was also the highest owing to its largest area, with the average value of 106.0 TgC/yr. The total NPP of evergreen needleleaf forest was the lowest, only 0.4 TgC/yr. The average values of total NPP of mixed broadleaf- needleleaf forest and deciduous needleleaf forest were higher, with the value of 92.7 TgC/yr and 58.6 TgC/yr, respectively.

The contribution of the different forest type’s NPP to total NPP in northeastern China was clearly different. The greatest was deciduous broadleaf forest, followed by mixed broadleaf- needleleaf forest and deciduous needleleaf forest. The smallest was evergreen needleleaf forest. NPP was contributed by various forest vegetation types found in the region, among them deciduous needleleaf forest, evergreen needleleaf forest, mixed broadleaf- needleleaf forest and deciduous broadleaf forest contributed 22.8%, 0.2%, 36.0%, and 41.2% of total NPP, respectively.

### 2. Spatial Patterns of NPP of Different Forest Types

**Figure 4:**
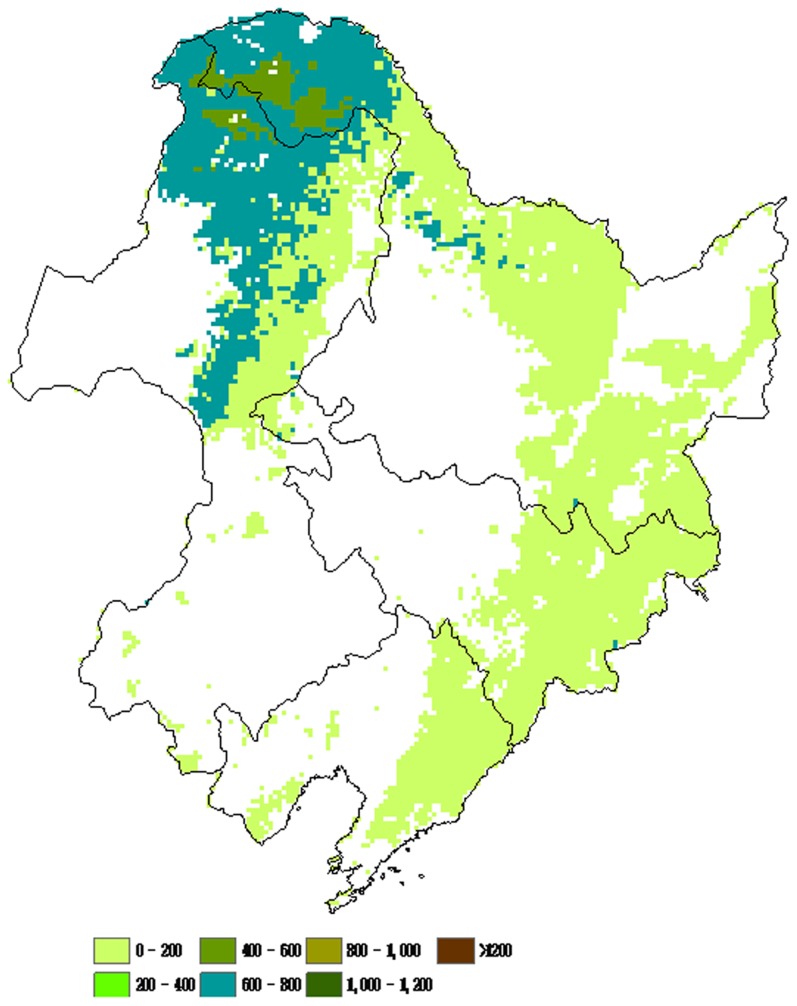
Spatial distributions of average NPP of deciduous needleleaf forest in northeastern China from 1981 to 2002 (g C/(m^2^/yr)).

**Figure 5:**
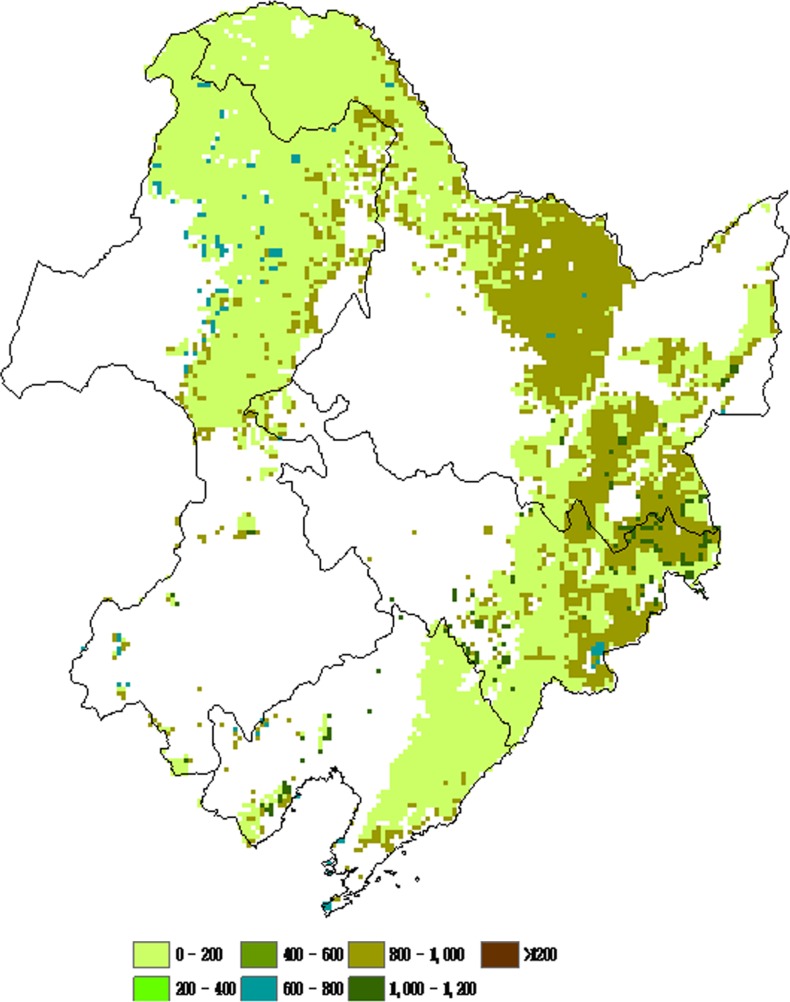
Spatial distributions of average NPP of mixed broadleaf-needleleaf forest in northeastern China from 1981 to 2002 (g C/(m^2^/yr)).

**Figure 6:**
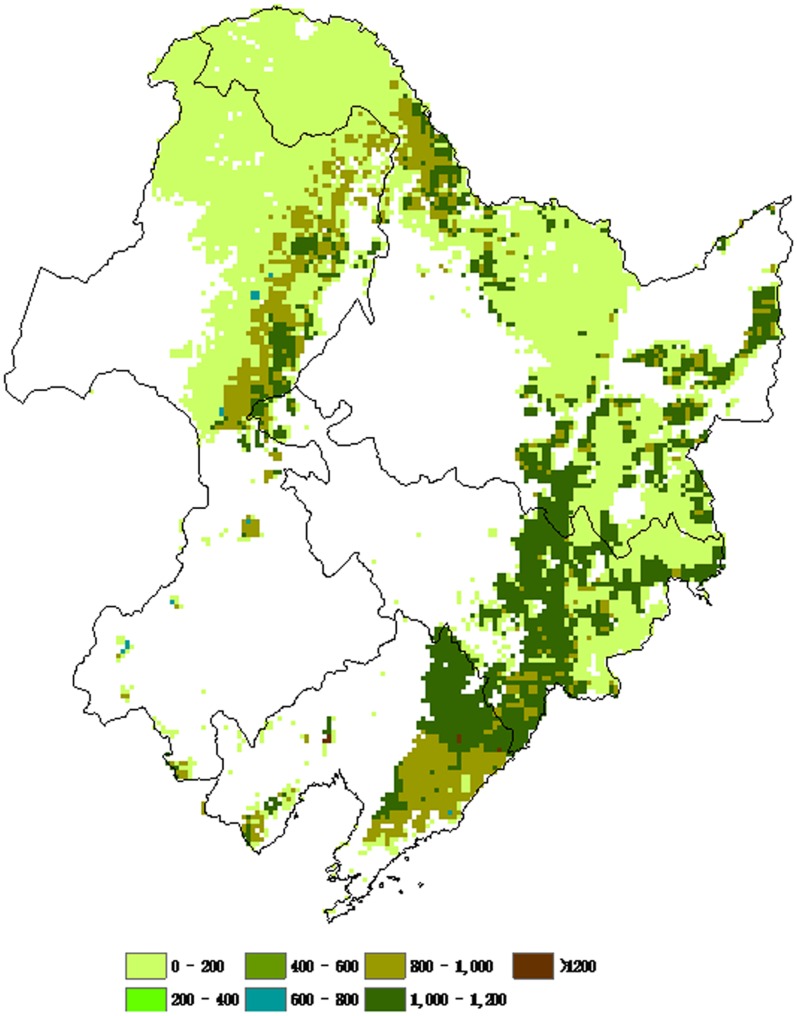
Spatial distributions of average NPP of deciduous broadleaf forest in northeastern China from 1981 to 2002 (g C/(m^2^/yr)).

**Figure 7:**
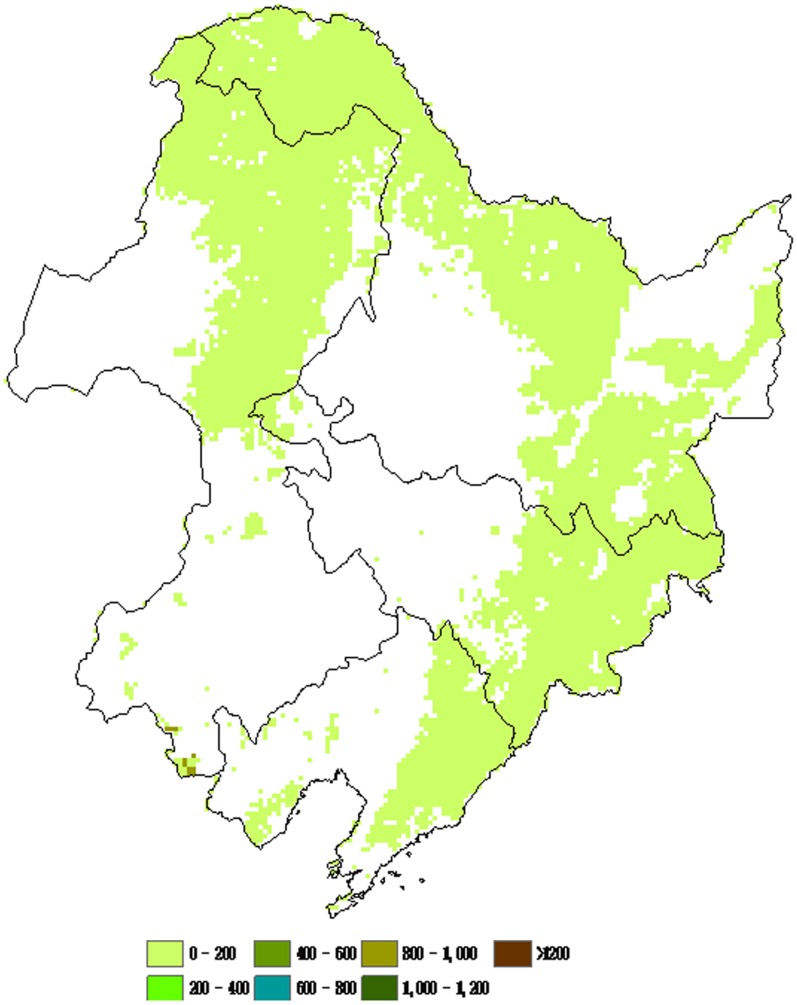
Spatial distributions of average NPP of evergreen needleleaf forest in northeastern China from 1981 to 2002 (g C/(m^2^/yr)).

The spatial distribution of NPP was associated with the vegetation cover and climate factors. The spatial patterns of forest NPP of different types in northeastern China were illustrated in Figure 4, Figure 5, Figure 6, and Figure 7. There was remarkable difference found in NPP between different forest types. Deciduous needleleaf forest was mainly distributed in the Daxing’anling Mountain range. Higher NPP values of deciduous needleleaf forest were found in the Daxing’anling Mountain (Figure 4). Mixed broadleaf- needleleaf forest with Higher NPP values located in the southeastern of Xiaoxing’anling and Jilin province (Figure 5). Higher NPP values of deciduous broadleaf forest were found in the Changbai Mountain (Figure 6). However, no remarkable difference in evergreen needleleaf forest NPP was found between regions (Figure 7), which was related with the variety’s biological characteristics of different forest types, and regional climatic conditions. The deciduous needleleaf forest in the Daxing’anling Mountain which was mainly distributed in high-altitude mountain, and could adapt to the cold, dry or humid climate, with strong continental climate. So, they had smaller autotrophic respiration and higher NPP. Mixed broadleaf- needleleaf forest in northeastern China was near the Japan Sea, with typical temperate maritime monsoon climate. However, due to the high latitude, the average annual temperature was low, and the summer was short and the winter was long. Thus, the autotrophic respiration of mixed broadleaf- needleleaf forest was also smaller and NPP was corresponding higher.

## Discussion

### 1. NPP of Different Forest Types

Net primary production (NPP) is a measure of plant biomass growth excluding respiration [28]. Thus, the NPP level is characteristic of the carbon accumulation rate. A related study for NPP by land cover showed that high NPP values occurred in forested areas, especially in the tropical and subtropical forest areas with warm climate and sufficient precipitation and radiation [29]. Forests in different regions within a biome also had different rates of NPP caused by many other site factors, including climate, soil, and drainage [30]. In our results, we found significant spatial-temporal changes of forest NPP in northeastern China due to variations. Our results indicated an increasing trend of forest NPP over the period, which was in agreement with previous studies [6], [14], [20]. High spatial heterogeneity was noted for NPP change within different forest types. Four reasons could contribute to such kinds of differences: climatic variables, age structure, species composition, and environmental factors. First, at continental to global scales, temperature and rainfall are the main factors that control variability in NPP [2], [31]. Second, different forest types have different age class compositions. Forest structure was critical factors determining forest ecosystem carbon storage and fluxes [7], [32], [33], [34], [35], [36]. Generally speaking, mature or maturing forests have higher NPP than young forests. Third, different forest types have different distribution patterns. At landscape, local to regional scales, especially in mountainous regions however, other environmental factors such as topography, geology, elevation, and soil type may play important roles in controlling the variability of NPP. This is one more possible reason for the discrepancy between forest productivity, also in China and in the globe.

### 2. Impacts of Climate Change on NPP of Different Forest Types

The changes in atmospheric CO_2_ concentration, temperature and precipitation regimes will likely affect the structure and distribution of boreal and temperate forests in northeast of China through their influences on forest regeneration, growth, mortality, physiological processes (e.g., photosynthesis, respiration) and ecological processes (e.g., the decomposition of soil organic materials). Such changes will result in a northward shift in the natural range of various forest types and species in this region. Quantifying the response of different vegetation types to climate change is vital, both nationally and regionally. Zhang and Yang investigated the climate–vegetation relationship and interaction for Chinese vegetation by using the Hodridge life-zone scheme [37]. Ni *et al*. adopted the terrestrial biosphere model of BIOME3 to predict the response of Chinese vegetation to future climate change scenarios for 2070–2099 projected by Hadley Centre coupled ocean–atmosphere GCM [38]. They found that a doubled CO_2_ climate shifts biomes north and west and climate change alone yielded a large reduction in boreal deciduous forest and a decline in desert, alpine tundra [38]. Wang *et al*. developed relationships between NPP and stand age for several major forest types in China using average NPP simulated with the BEPS model driven by remote sensing inputs and forest age obtained from inventory data [39]. They pointed out that these relationships were highly significant, and the patterns of NPP variation with stand age were similar for different forest types [39].

Generally speaking, the primary factors controlling the carbon cycle of vegetation include temperature, precipitation, phenological state, carbon dioxide concentration in the atmosphere, *etc*. Temperature increase can result in two aspects′ effects on the productivity of the ecosystem in the northern mid-to-high latitude ecosystems [12]. Firstly, the positive effect is that temperature increase might increase the length of the growing season of vegetation, improving photosynthesis efficiency, and enhancing the productivity of vegetation [12], [40]. Secondly, the negative effect may be that temperature increase can increase the consumption of water and bring on a water deficit in some biomes [41]. Moreover, the phenological state of vegetation significantly affects exchanges of carbon dioxide and water between the Earth’s surface and the atmosphere. Especially in the high latitudes, solar radiation and temperature are highly seasonal, showing a mix of photoperiod and temperature limits. Jolly *et al*. pointed out that phenology is co-limited by multiple factors in many locations, but these co-limitations have never been expressed spatially for the globe [42]. In this study, the interannual increases in NPP of different forest types in northeastern China from 1981 to 2002 were mainly caused by the phenological state.

However, it is an important and uncertain issue whether or not carbon dioxide in atmosphere has a long-term impact on forest terrestrial productivity [12], [43]. The growth enhancement from CO_2_ enrichment generally occurs through increases in the rates of net photosynthesis in the order of 40%–80%, compounded by an increase in leaf area, while observed long-term increases in net photosynthesis are typically somewhat lower than the short-term response [44]. The downward acclimation with time of photosynthesis appears to be related primarily to dilution of the leaf N concentration, and CO_2_ enhancement of forest productivity is constrained by limited nitrogen availability [45]. Based on recent research progresses and with consideration of the complexity and uncertainty of the carbon dioxide fertilization effect, our current simulation did not consider the effect of carbon dioxide on the NPP forests. Meteorological elements in FORCCHN model, such as radiation, precipitation, and temperature, etc, are basic driving force in the process of forest carbon sequestration, and therefore coupling FORCCHN model with climate models will be more objective to quantitative the response of forest carbon absorption capacity to climate change. These complex processes and interactions will be addressed in the next version of the model while climate models are coupled.

### 3. Limitations

The carbon budget model FORCCHN was established based on individual tree species. In this study, we focused on the different forest types. And the FORCCHN model had been improved and intensively tested with various field measurements in northeastern China [12].The results presented here represent our first attempt to apply the improved FORCCHN model for studying the spatial-temporal distributions of NPP of different forest types (evergreen broadleaf forest?deciduous broadleaf forest?evergreen needleleaf forest?deciduous needleleaf forest) in northeastern China from 1981 to 2002. Our simulation results suggested that there were obvious spatial-temporal variations between different forest types. Unfortunately, because of the current limitation on the large-scale simulation of domestic forest carbon cycle, and the difficulty and accuracy in obtaining data of model input, there still existed some unsolved problems and limitations in our results. Firstly, as far as the spatial data were concerned, the interpolation accuracy and quality of soil data were to be improved. In this study, the soil data was from the second national soil survey profile data and other information (including the local soil records, literature, etc.). Measurement specification of data from different sources might be quite different. So, during the result analysis, the error of data source itself could not be ignored. The error of data in estimating carbon dynamics could be significantly reduced by increasing the sample data points [39], [46], and therefore, we call for more ground-based NPP measurements. However, there were many difficulties and could not be quantified analysis in realistic analysis and evaluation of these errors. Moreover, these data sources were discrete distribution in space, affecting the accuracy while interpolated into the other points and matching between different spatial data. Meteorological data used in this study were from the meteorological observation specification data with higher accuracy. Therefore, the impact caused by the meteorological data source on simulation result was not obvious.

Secondly, the existing formulation of FORCCHN had no fire disturbance submodel and was unable to explicitly predict the impacts of fire disturbances on forest NPP and carbon budgets. Moreover, extreme weather events, pests and diseases were additional weaknesses of the model, because these factors were important for affecting forest growth and development. The extreme events might counteract the effects of the anticipated mean warming and lengthening of the growing season, and reduce the productivity of ecosystems, reversing sinks to sources. The increasing climate stress (e.g. more intense, more frequent, and longer lasting heat waves and droughts) seemed likely to increasingly reduce NPP and carbon sequestration over the next century [47], [48], [49].

Finally, the limitations of model parameter and initial condition might cause potential limitations in modeling NPP of different forest types, because the real situation was not entirely the same with the simulation. Moreover, we did not take into accounting the relationships between soil and forest vegetation types. These limitations presented here had potential impact on the accuracy of the simulation results.

### Conclusions

The results presented in this paper show that distribution and change of forest NPP in northeastern China were related with the different forest types. From 1981 to 2002, NPP of different forest type in northeastern China exhibited interannual increase trends, showing larger NPP fluctuation difference. This would improve projections of future forest resource development, with clear implications for carbon sequestration of different forest types. The contribution of the different forest type’s NPP to total NPP in northeastern China was clearly different, and the greatest was deciduous broadleaf forest, followed by mixed broadleaf- needleleaf forest, deciduous needleleaf forest and evergreen needleleaf forest. Moreover, there was remarkable spatial difference in NPP between different vegetation types. Higher NPP values of deciduous needleleaf forest, mixed broadleaf- needleleaf forest and deciduous broadleaf forest were found in the Daxing’anling region, the southeastern of Xiaoxing’anling and Jilin province, and the Changbai Mountain, respectively. However, No regional differences were found for evergreen needleleaf forest NPP. In summary, this study provides further evidence of increasing NPP in northeastern China. The exact dynamics of these changes require further investigation with both modeling and field-based studies.
